# The *Saccharomyces cerevisiae* Telomerase Subunit Est3 Binds Telomeres in a Cell Cycle– and Est1–Dependent Manner and Interacts Directly with Est1 *In Vitro*

**DOI:** 10.1371/journal.pgen.1002060

**Published:** 2011-05-05

**Authors:** Creighton T. Tuzon, Yun Wu, Angela Chan, Virginia A. Zakian

**Affiliations:** Department of Molecular Biology, Princeton University, Princeton, New Jersey, United States of America; Fred Hutchinson Cancer Research Center, United States of America

## Abstract

Telomerase is a telomere dedicated reverse transcriptase that replicates the very ends of eukaryotic chromosomes. *Saccharomyces cerevisiae* telomerase consists of TLC1 (the RNA template), Est2 (the catalytic subunit), and two accessory proteins, Est1 and Est3, that are essential *in vivo* for telomerase activity but are dispensable for catalysis *in vitro*. Est1 functions in both recruitment and activation of telomerase. The association of Est3 with telomeres occurred largely in late S/G2 phase, the time when telomerase acts and Est1 telomere binding occurs. Est3 telomere binding was Est1-dependent. This dependence is likely due to a direct interaction between the two proteins, as purified recombinant Est1 and Est3 interacted *in vitro*. Est3 abundance was neither cell cycle–regulated nor Est1-dependent. Est3 was the most abundant of the three Est proteins (84.3±13.3 molecules per cell versus 71.1±19.2 for Est1 and 37.2±6.5 for Est2), so its telomere association and/or activity is unlikely to be limited by its relative abundance. Est2 and Est1 telomere binding was unaffected by the absence of Est3. Taken together, these data indicate that Est3 acts downstream of both Est2 and Est1 and that the putative activation function of Est1 can be explained by its role in recruiting Est3 to telomeres.

## Author Summary

Owing to the biochemical properties of DNA polymerases, the free ends of linear chromosomes, called telomeres, cannot be replicated by the same mechanisms that suffice for the rest of the chromosome. Instead they are maintained by a telomere-dedicated reverse transcriptase called telomerase that uses its integral RNA component as the template to make more telomeric DNA. In baker's yeast, telomerase is composed of a catalytic subunit (Est2), the templating RNA (TLC1), and two accessory proteins, Est1 and Est3. Here we show that Est3 associates with telomeres late in the cell cycle, at the same time when telomerase is active, and this binding was Est1-dependent, even though Est3 abundance was neither cell cycle–regulated nor Est1-dependent. Since purified Est3 and Est1interacted *in vitro*, Est1-dependent recruitment of Est3 is probably due to direct protein–protein interaction. Neither Est1 nor Est2 telomere binding was Est3-dependent. Thus, Est3 acts downstream of telomerase recruitment to promote telomerase activity, and the telomerase activation functions of Est1 can be explained by its recruiting Est3 to telomeres.

## Introduction

Telomeres, the DNA-protein structures at the ends of most eukaryotic chromosomes are essential for genome integrity: they protect chromosomes from degradation and end-to-end fusions, distinguish chromosome ends from DNA breaks, position chromosomes for pairing in meiosis and ensure the complete replication of chromosome ends (reviewed in [1]–[3]). Telomeric sequences are comprised of highly repetitive DNA in which the strand running 5′ to 3′ towards the chromosome end is G-rich and extended to form a 3′ single stranded tail. For example, throughout most of the cell cycle, each *Saccharomyces cerevisiae* telomere has ∼300 bps of duplex C_1-3_A/TG_1-3_ DNA ending with a short ∼12–14 base TG_1-3_-tail [4].

In most eukaryotes, a specialized reverse transcriptase called telomerase provides the basis for an RNA templated replication mechanism that elongates the G-rich strand of telomeric DNA. The *S. cerevisiae* telomerase consists of the catalytic reverse transcriptase subunit, Est2 [5], the templating RNA component, TLC1 [6], and two regulatory proteins Est1 [7] and Est3 [8], [9]. Eliminating any one of these four gene products results in the *est* (ever shorter telomeres) phenotype, characterized by gradual telomere shortening and death in most cells after ∼50–100 generations [6]–[8]. In addition, certain alleles of *CDC13*, such as *cdc13-2*, a gene that encodes an essential protein that binds the 3′ single stranded TG_1-3_ tails *in vivo* [10], [11], are telomerase defective [12]. Cdc13 is a multi-functional telomere binding protein that is essential to protect chromosome ends from degradation [13] and has a key function in telomerase recruitment [14], [15].

Most of the yeast telomere is replicated by standard semi-conservative DNA replication, which occurs at the end of S phase [16], [17]. This replication is followed by C-strand resection, which generates long (∼50–100 base) transient single-stranded G-tails [17]–[19]. Telomerase action is also restricted to late S/G2 phase [20], [21], even though Est2 is telomere associated throughout most of the cell cycle with peak binding in both G1 and late S/G2 phase [22]. Est2 telomere association during G1 and early S phase requires a specific interaction between TLC1 and the heterodimeric Ku complex [23]. Est2 telomere association in late S/G2 phase is low in *cdc13-2* cells [22], requires a specific interaction between a stem-bulge region on TLC1 RNA and Est1 [24], and is lost entirely in *tlc1Δ* cells [22], [24]. Est1 telomere binding, which occurs only in late S/G2 phase, coincident with telomerase action [22], is low when it cannot interact with TLC1 RNA or in *cdc13-2* cells and is eliminated altogether in *est2Δ* cells [24]. Moreover, Est1 abundance is cell cycle regulated, low in G1 and early S phase, and peaking in late S/G2 phase [22], [25]

Although both Est1 and Est3 are essential for telomerase action *in vivo* [8], the requirement for Est1 (but not Est3) can be bypassed by expressing a DBD_Cdc13_-Est2 fusion protein (DBD, DNA binding domain) [14]. This result is consistent with a model in which a Cdc13-Est1 interaction recruits the telomerase holoenzyme to the telomere, an interpretation supported by biochemical and genetic data that show that the two proteins interact *in vivo* [26], [27]. However, Est1 has a role other than recruitment as it is needed for the hyper-elongation of telomeres that occurs in cells expressing a DBD_Cdc13_-Est2 fusion [14]. This extra function can be seen *in vitro* as well: Est1 is required for long extension products in a PCR based *in vitro* assay [28], and its addition to a primer extension assay increases the amount of product [29]. In *Candida albicans*, Est1 affects both initiation and processivity of telomerase *in vitro* in a primer-specific manner [30]. Thus, Est1 appears to function in both recruitment and activation of telomerase.

The telomeric role of Est3 is separable from that of Est1 as an Est3-DBD_Cdc13_ fusion cannot bypass the requirement for Est1 and an Est1-DBD_Cdc13_ fusion cannot rescue the telomerase defect of an *est3*Δ strain [9]. Nonetheless, the two proteins are interconnected. In *C. albicans*, Est3 and Est1 mutually depend on each other for assembly into the telomerase holoenzyme [30]. However, the situation in *S. cerevisiae* is unclear as using co-immunoprecipitation, one group found that Est3 association with Est2/TLC1 is Est1 dependent [25] while one did not [9], [31]. *In vitro*, extracts prepared from a *C. albicans est3Δ* strain show the same initiation and processivity defects in telomerase assays as extracts from *est1Δ* cells [30], while all primers are extended less efficiently in extracts from an *est3Δ S. castellii* strain [31].Est3 from both *S. cerevisiae* and *C. albicans* has structural similarity to TPP1 within an OB-fold domain [32], [33], a mammalian telomere structural protein that has roles in both telomere end protection and promoting telomerase activity [34]–[36].

Here we used chromatin immuno-precipitation (ChIP) in mutant and WT cells to determine the temporal pattern and genetic dependencies for *S. cerevisiae* Est3 telomere binding. We show that Est3 telomere binding occurred mainly in late S/G2 phase and was at background or close to background levels in *tlc1Δ*, *est1*Δ and *est2Δ* cells. In contrast, the late S/G2 phase association of both Est1 and Est2 was not reduced in *est3*Δ cells, making *est3Δ* the first telomerase deficient strain where the temporal and quantitative pattern of Est2 telomere binding is indistinguishable from that in WT cells. As purified Est1 and Est3 interact *in vitro*, the putative activation role of Est1 can be explained by its role in recruiting Est3 to telomeres. We also determined the absolute copy number for each of the three Est proteins, the first such determination for any protein subunit of telomerase in fungi.

## Results

### Est3-G8-Myc18 telomere binding occurs mainly in late S/G2 phase, and its abundance is not cell cycle–regulated

**Figure 1:**
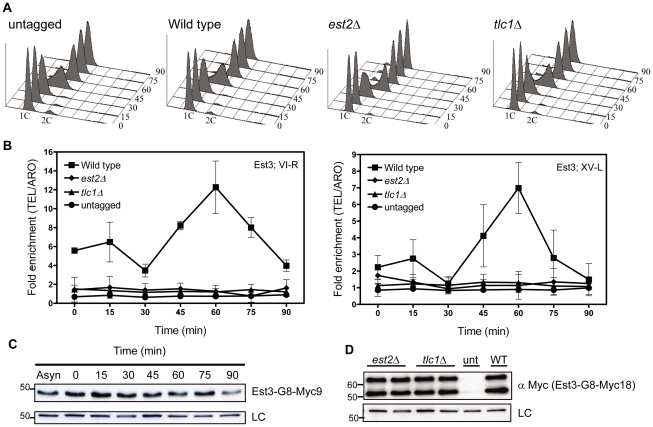
Est3 telomere binding is biphasic but highest in late S/G2 phase. Strains expressing *EST3-G8-MYC18* were arrested with α factor and then released into the cell cycle. Samples were collected for FACS and ChIP analysis at the indicated times. Fixed cells were immuno-precipitated with anti-Myc antibody and purified DNA was analyzed by real-time PCR. Data are represented as the fold enrichment of telomeric DNA (VI-R or XV-L) relative to the non-specific locus (*ARO1*) after normalization to input DNA. Error bars represent one standard deviation from four biological replicates. (A) Representative FACS analysis from one of four synchronies from untagged, *est2*Δ, *tlc1*Δ and WT strains expressing Myc-tagged Est3. (B) Quantitative real-time PCR analysis of Myc-tagged Est3 telomere association. In *est2*Δ cells, the amount of telomere associated Est3 at late S/G2 phase was significantly reduced from WT at both telomeres (VI-R, P = 0.0052; XV-L, P = 0.0012). In *tlc1*Δ cells, the late S/G2 phase (60 min) telomere association of Est3 was significantly reduced from WT at both telomeres (VI-R, P = 0.0078; XV-L, P = 0.0085). (C) Anti-Myc Western blots of protein extracts from synchronous cells expressing Est3-G8-Myc9 (upper panel). Here and in subsequent figures the loading control (LC) was α-tubulin. (D) Protein extracts from asynchronous cells expressing Myc-tagged Est3 analyzed by western blot using anti-Myc antibody in *est2Δ*, *tlc1Δ*, untagged (unt) and WT (WT) strains. Est3-G8-Myc9 and Est3-G8-Myc18 migrated more slowly than their predicted molecular masses probably due to their acidic nature. A single band of ∼45 kDa was detected by western analysis in extracts from *EST3-G8-MYC9* strains (panel C), while two bands of ∼55 kDa and ∼65 kDa were often (panel D) detected in *EST3-G8-MYC18* extracts. We infer that the ∼65 kDa species corresponded to the full-length protein while the ∼55 kDa band was probably an N-terminal degradation product, as the smaller ∼55 kDa protein was barely detectable in some extracts (Figure S1C).

As in previous work from our lab, we used chromatin immuno-precipitation (ChIP) to determine protein association with telomeres *in vivo* (e.g. [22]). Previous studies from other labs used an HA3-tagged version of Est3 [9], [25] to study its association with other telomerase subunits, but this protein was not detectable at telomeres by ChIP (our unpublished results). Est3 directly tagged with nine Myc-epitopes was not functional (data not shown). Therefore, we epitope tagged Est3 at its carboxyl-terminus with a glycine linker (G8), which improves the functionality of epitope tagged proteins [37], followed by either 9 or 18 Myc epitopes. As with all of the epitope tagged proteins used in this paper, Est3 was expressed from its own promoter as the only copy of *EST3* in the strain. Cells expressing these Est3 alleles did not senesce and maintained stable telomere length, although as in the HA3-tagged strain [9], [25], telomeres were shorter than in WT cells (see methods and Figure S1A for more details). Both Myc-tagged proteins were detectable by an anti-Myc antibody in western blotting of whole cell extracts (Figure 1C, Figure S1B), but only Est3-G8-Myc18 gave reliable results in a ChIP assay.

**Figure 2:**
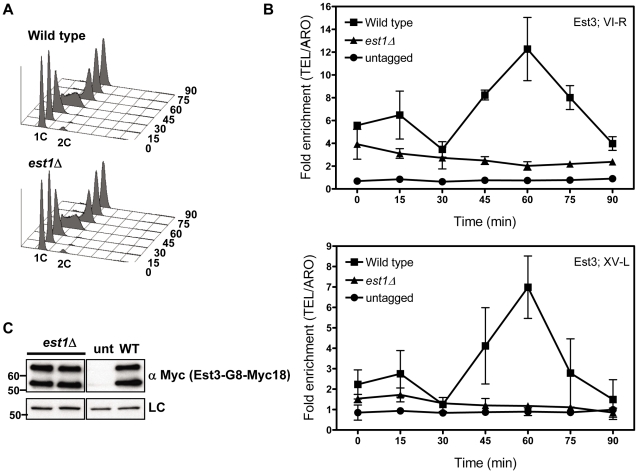
Telomere association of Est3 at late S/G2 phase is reduced in the absence of *EST1*. Methods are the same as for Figure 1. (A) Representative FACS analysis from one of four biological replicates from *est1*Δ and WT strains expressing Myc-tagged Est3. (B) Quantitative real-time PCR analysis of Myc-tagged Est3 telomere association from untagged, WT and *est1*Δ cells. The amount of telomere associated Est3 at G1 phase in *est1*Δ cells was low but still significantly higher than in untagged cells at TEL-VI-R (0 min, P = 0.024; 15 min, P = 0.002; 30 min, P = 0.023) and at TEL-XV-L (0 min, P = 0.044; 15 min, P = 0.035). At early S-phase (30 min), Est3 telomere association was significant in *est1*Δ cells compared to the untagged strain at TEL-VI-R (P = 0.023) but not at TEL-XV-L (P = 0.098). In *est1*Δ cells, the amount of telomere associated Est3 at late S/G2 phase was significantly reduced at TEL VI-R (60 min, P = 0.0007) and at TEL XV-L (60 min, P = 0.0028) compared to WT. The amount of telomere associated Est3 later in the cell cycle was significant compared to the untagged strain at TEL-VI-R (45 min, P = 0.02; 60 min, P = 0.04; 75 min, P = 0.03; 90 min, P = 0.04) but not at TEL-XV-L (45 min, P = 0.09; 60 min, P = 0.07; 75 min, P = 0.46; 90 min, P = 0.58). (C) Anti-Myc western blots from whole cell protein extracts from asynchronous *est1*Δ, untagged (unt) and WT (WT) strains expressing Myc-tagged Est3.

We used real-time PCR quantitation to evaluate the association of Est3-G8-Myc18 to two native telomeres, the right arm of chromosome VI (TEL-VI-R) and the left arm of chromosome XV (TEL-XV-L) throughout a synchronized cell cycle (Figure 1, Figure 2). For all synchrony experiments, cells were arrested in late G1 phase with alpha factor and then released into the cell cycle. The quality of each synchrony was evaluated by flow cytometry, which revealed no major reproducible differences in cell cycle progression among the various strains used in this study (Figure 1A, Figure 2A). We used real-time PCR to determine the amount of telomeric DNA in the immuno-precipitate. Synchronies were done at least three times with the data presented as the average telomere association +/− one standard deviation. At each time point, we normalized the telomeric signal to the signal at the non-telomeric *ARO1* locus in the same sample.

At both telomeres, the profile of Est3 telomere association in WT cells was bi-phasic, with a small peak in G1 phase (0 and 15 min) and 2-2.5-fold higher binding in late S/G2 phase (60 min) (Figure 1B). This late S/G2 binding was ∼10-fold above the no tag control. This biphasic binding pattern was reminiscent of Est2 telomere association except that for Est2, the peaks in G1 and late S/G2 phases were of similar magnitude [22]. Compared to the untagged strain, the G1 telomere binding was significant at TEL-VI-R (P = 0.020) but not at TEL-XV-L (P = 0.078), while the late S/G2 phase Est3-G8-Myc18 association was significant at both telomeres (P = 0.0086, TEL-VI-R; P = 0.0079, TEL-XV-L). Differential binding throughout the cell cycle was not due to cell cycle variations in protein abundance as levels of tagged Est3 were constant throughout the cell cycle (Figure 1C; Figure S1C.). We conclude that the telomere association of Est3 occurs mainly at late S/G2 phase, which coincides temporally with the peak of Est1 telomere association, the second peak of Est2 telomere binding, and the time of telomerase action (see Introduction).

### Est3-G8-Myc18 telomere binding is Est2-, TLC1-, and Est1-dependent

Next, we determined if Est3-G8-Myc18 telomere binding requires the presence of other telomerase components by examining its telomere association in synchronized *est2*Δ, *tlc1*Δ (Figure 1B), and *est1*Δ (Figure 2B) cells. In the absence of either Est2 or TLC1 RNA, Est3-G8-Myc18 telomere association was not detected at any point in the cell cycle at either TEL-VI-R or TEL-XV-L (see legend of Figure 1 and Figure 2 for P values). The reduced Est3 telomere binding in *est2*Δ and *tlc1*Δ cells was not due to reduced Est3 abundance (Figure 1D). Therefore, both Est2 and TLC1 RNA are absolutely required for Est3 telomere association.

In *est1*Δ cells, the amount of telomere associated Est3-G8-Myc18 at late S/G2 phase was significantly reduced at TEL VI-R (60 min, P = 0.0007) and at TEL XV-L (60 min, P = 0.0028) compared to the level of binding in WT cells (Figure 2B). When compared to the no tag control, the level of Est3-G8-Myc18 binding from 45 to 75 minutes at telomere VI-R was low but still significant while the level of binding to XV-L was not significant (see Figure 2 legend for P values for each time point). Est3-G8-Myc18 association in G1 and early S phase (Figure 2B, 0 to 30 min) was also reduced but was significantly higher than in the no tag control at both telomeres (see Figure 2 legend for P values). Although there was a small amount of Est3-G8-Myc18 telomere binding in the absence of Est1, Est3 telomere association was largely Est1 dependent.

### Purified Est3 and Est1 interact *in vitro*

**Figure 3:**
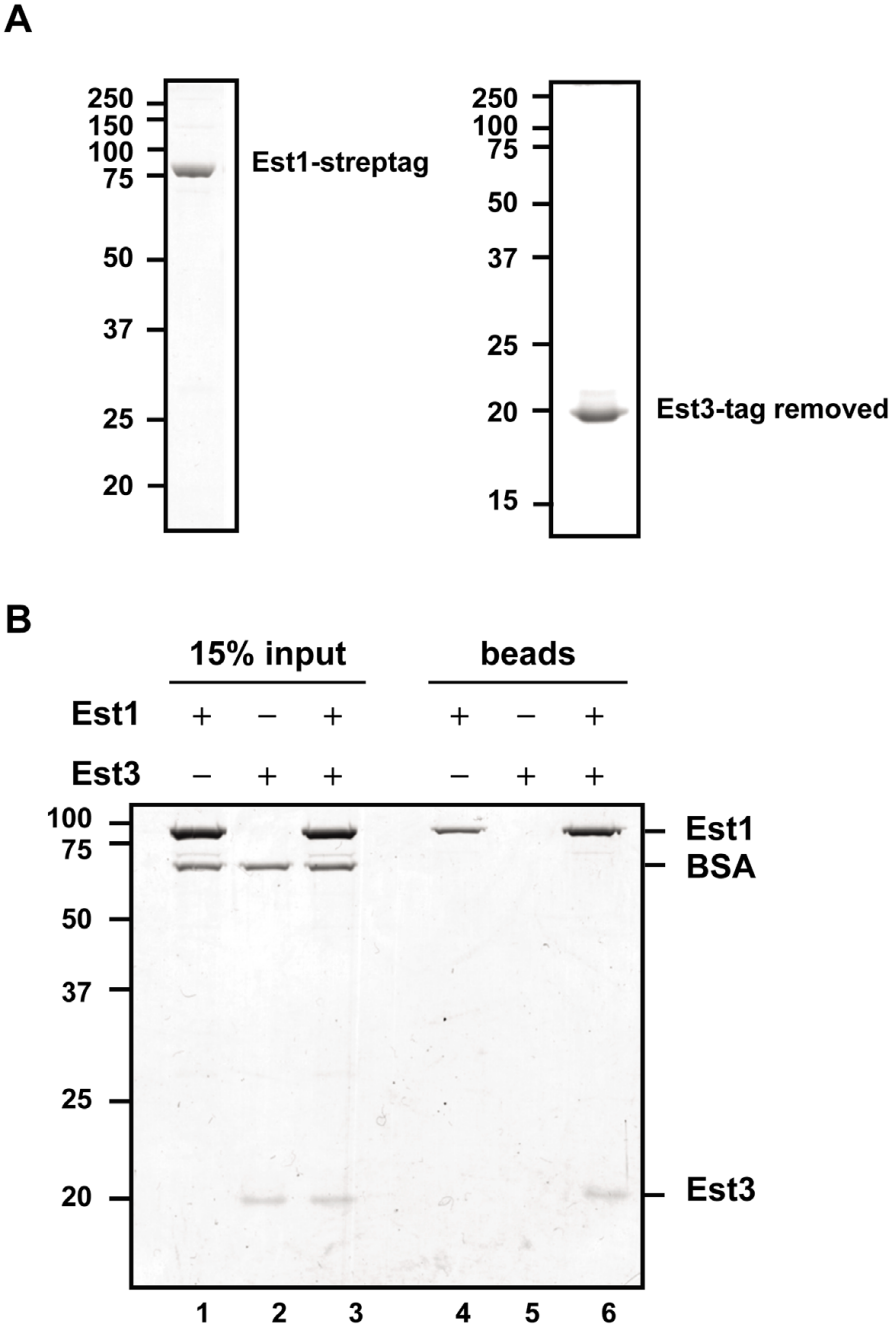
Est1 and Est3 interact directly *in vitro*. (A) 1 µg of purified Strep-tagged Est1 (left panel) and tag-removed Est3 (right panel) was resolved on SDS-PAGE gels followed by staining with Coomassie brilliant blue. (B) Interaction between purified Est1 and Est3 was assessed by magnetic beads pull-down assay. The beads fractions is shown in lanes 4-6 (“beads”), and 15% of the input materials is shown as a control in lanes 1–3 (“15% input”). Positions of Est1, BSA, and Est3 are indicated on the right. Proteins were separated on a 10% SDS-PAGE gel and visualized by Coomassie blue staining.

Est1 could affect Est3 telomere binding directly or indirectly. To distinguish between the two possibilities, we purified C-terminally strep-tagged [38] Est1 from *S. cerevisiae* and N-terminally strep-tagged Est3 from *E. coli*, and removed the Est3 strep-tag post-purification. Both proteins were purified to near homogeneity (Figure 3A), and their identities verified by MS/MS mass spectrometry. When expressed from its own promoter on a *CEN* plasmid as the only copy in the cell, strep-tagged Est1 supported WT length telomeres (data not shown). We tested the ability of the two purified proteins to interact using a magnetic bead pull down experiment (Figure 3B). Purified Est1 was mixed with streptavidin-coated magnetic beads that capture the C-terminal affinity tag of Est1 (lane 4). Est3 was not pulled down by the beads in the absence of Est1 (lane 5). However, in the presence of Est1, Est3 was bead-associated (lane 6). BSA, which was used as a negative control, was not bead associated either in the presence or absence of Est1. Further evidence for specificity is provided by similar assays where Est1 did not interact with the DBD region of Cdc13, and Est3 did not interact with full length Cdc13 (YW and VAZ, in preparation).

### The late S/G2 phase association of Est1 and Est2 is not reduced in est3Δ cells

**Figure 4:**
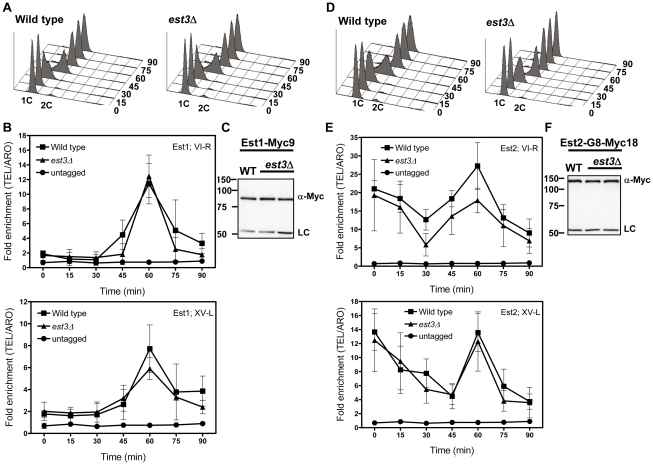
The late S/G2 phase telomere association of Est1 and Est2 is not reduced in the absence of *est3*Δ. Methods are the same as for Figure 1. (A) Representative FACS analysis from one of three biological replicates from WT and *est3*Δ cells expressing Myc-tagged Est1. (B) Quantitative real-time PCR analysis of Myc-tagged Est1 telomere association. The amount of telomere associated Est1 at late S/G2 phase was indistinguishable between WT and *est3*Δ cells. (C) Anti-Myc western blot analysis from whole cell protein extracts from asynchronous cultures expressing Myc-tagged Est1. (D) Representative FACS analysis from one of three biological replicates from WT and *est3*Δ cells expressing Myc-tagged Est2. (E) Quantitative real-time PCR analysis of Myc-tagged Est2 telomere association. The amount of telomere associated Est2 at late S/G2 phase was indistinguishable between WT and *est3*Δ cells throughout the cell cycle at both telomeres except at 30 min (p = 0.05) for TEL VI-R. (F) Anti-Myc western blot analysis of whole cell protein extracts from asynchronous cells expressing Myc-tagged Est2.

As monitored by whole cell and immuno-precipitate western experiments [22], [23], [25], [39], Est1 abundance is cell cycle regulated, low in alpha factor arrested G1 phase cells and peaking at late S/G2 phase. Consistent with our previous studies and coincident with its peak in abundance, Est1 telomere binding occurred at late S/G2 phase (60 min) at both telomeres in WT cells (Figure 4B). In *est3*Δ cells, Est1 binding at TEL-VI-R was indistinguishable from WT (60 min, P = 0.68). Est1 association at TEL-XV-L was marginally lower in *est3*Δ compare to WT cells (60 min), but this difference was not significant (P = 0.26). Est1 abundance was also not Est3 dependent (Figure 4C). We conclude that the telomere association of Est1 is Est3 independent.

Likewise, Est2 telomere binding (Figure 4E) and its abundance (Figure 4F) were very similar in WT and *est3Δ* cells. As shown previously [22], in WT cells, Est2 bound to TEL-VI-R and TEL-XV-L throughout the cell cycle with peak binding in G1 (0, 15 min) and late S/G2 phases (60 min) (Figure 4E). In *est3*Δ cells, Est2 binding was indistinguishable from WT throughout the cell cycle at both telomeres except at 30 min (p = 0.05) at TEL VI-R. We conclude that the telomere association of the catalytic subunit Est2 is also Est3 independent.

### Phosphorylation of Est1, Est2, or Est3 was not detected

Telomerase action is cell-cycle regulated [20], [21], and Cdc13 and each of the three Est proteins binds telomeres in a cell cycle dependent manner ([22] and Figure 1). Nonetheless, Est1 is the only one of these proteins whose abundance is cell cycle regulated [22], [25]. The checkpoint kinase Tel1 binds telomeres in a cell cycle dependent manner, and this binding is necessary for preferential association of Est2 and Est1 with a short VII-L telomere [40]. Cdk1/Cdc28 activity is required for C-strand degradation [41], [42], and Cdc13 is phosphorylated by Cdk1 in a cell cycle dependent manner that affects the level of Est1 telomere association [43], [44]. Since Est1, Est2 and Est3 contain one or more candidate Tel1 and Cdk1 phosphorylation sites, one possibility is that their cell cycle regulated telomere binding is due to cell cycle regulated phosphorylation. However, there is no evidence for slower migrating species for any of the three Est proteins when analyzed by conventional polyacrylamide gels ([22] and Figure 1, Figure 4).

**Figure 5:**
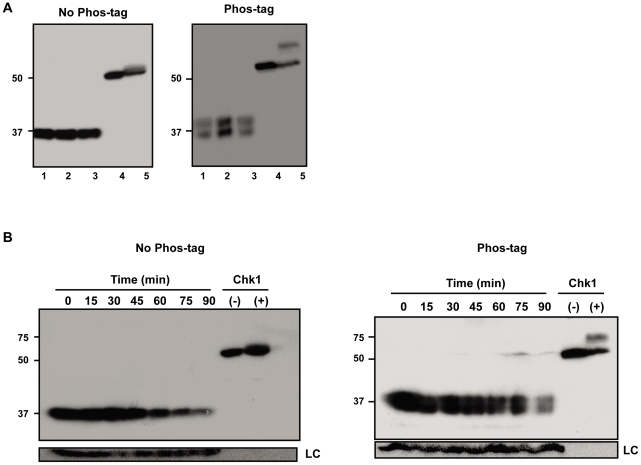
A fraction of Est3 appears to be phosphorylated, but this behavior is not cell cycle–regulated. Extracts from cells expressing Est3-G8-Myc9 were separated in 10% SDS-PAGE gels without (No Phos-tag, left panel) or with 100 µM phos-tag (Phos-tag, right panel). (A) Samples from three different asynchronous cultures. (B) Extracts taken throughout a synchronous cell cycle from a representative synchrony (of six total such experiments). Lanes labeled Chk1 contain extract from asynchronous *S. pombe* cells expressing 3HA-Chk1 where the cells were either treated (+) or not (−) with 40 µM camptothecin *in vivo*. Chk1, which is phosphorylated in response to DNA damage [62] serves as a positive control for the ability to detect phosphorylated proteins with the Phos-tag method.

To address in more detail the possibility that Est proteins are phosphorylated, we prepared protein extracts from synchronized cells expressing either Est1-Myc9, Myc9-Est2, or Est3-G8-Myc9 and separated the extracts in gels containing Phos-tag (Wako), a reagent that binds phosphate groups resulting in slower mobility of phosphorylated proteins [45]. In the extracts containing Est1-Myc9 or Myc9-Est2, there was no detectable fraction of the protein with reduced mobility in Phos-tag gels (Figure S2). This pattern was seen for extracts resolved in 25 µM Phos-tag (as shown in Figure S2) as well as in 100 µM phos-tag (data not shown). Likewise, the mobility of Est3-G8-Myc9 was not affected by 25 or 50 µM Phos-tag (data not shown). However, extracts from cells expressing Est3-G8-Myc9 and resolved in gels containing 100 µM Phos-tag had about equal amounts of a slower migrating form of Est3 (Figure 5, right panels) that was not detected in the absence of Phos-tag (Figure 5, left panels). The two species were of similar levels in extracts from asynchronous (Figure 5A), G1 arrested (Figure 5B, 0 min) or from throughout a synchronous cell cycle (Figure 5B, 15–90 min). Thus, the apparent phosphorylation of Est3 revealed in the presence of Phos-tag was not cell cycle regulated.

### Est1, Est2, and Est3 are all low abundance proteins

**Figure 6:**
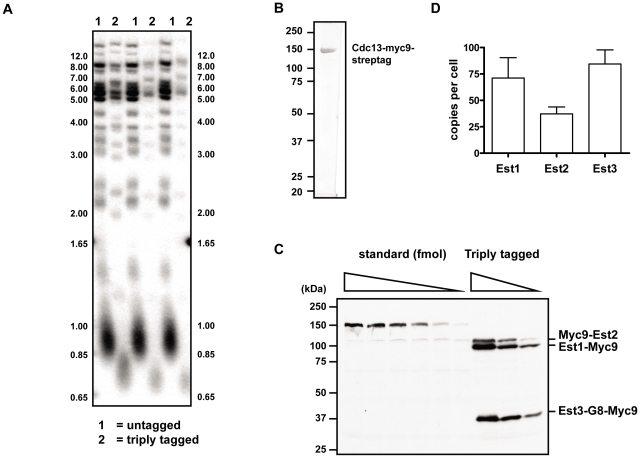
*In vivo* concentration of telomerase components. (A) Telomere length analysis in strain where each of the three Est proteins is tagged with Myc9 epitopes (lanes labeled 2) or in the untagged parental strain (lanes labeled 1). Three independent clones from each of the triply tagged strain and an isogenic untagged strain were chosen for telomere Southern blot analysis. Strains were cultured at 30°C in rich media. Genomic DNA was digested with *Pst*I and *Xho*I, separated in a 1% TBE agarose gel, and transferred to Hybond N+ nylon membrane (GE Healthcare). Blots were hybridized to a randomly primed ^32^P-labeled gel purified telomere fragment from *EcoR*I digested pCT300 [63] and exposed to a phosphorimager for analysis. (B) 1 µg of purified standard protein, Cdc13-myc9-streptag, was resolved on SDS-PAGE gel followed by staining with Coomassie brilliant blue. (C) A representative gel of quantitative western blot analysis. Proteins were separated in a 9% SDS-PAGE gel and blotted with anti-Myc antibody. (D) Quantification of *in vivo* concentrations of the Est protein components. Error bars represent one standard deviation from average of three independent experiments.

As part of our efforts to understand Est protein function, we generated Myc9-tagged versions of each of the three Est proteins (here and [23]). We used these tagged alleles to generate a strain in which Est1, Est2, and Est3 were each marked with nine Myc epitopes. The triply tagged strain maintained stable telomeres that were ∼75–125 bps shorter than WT telomeres (Figure 6A) and showed no evidence of senescence even after >6 restreaks (data not shown).

We measured the absolute abundance of each of the Myc9-tagged proteins using quantitative western blot analyses. In order to provide a standard to convert western signals to absolute protein levels, a Myc9-tagged Cdc13 protein was fused to a C-terminal tandem 5X Strep-Tag II, over-expressed in *S. cerevisiae*, and purified to homogeneity (Figure 6B). Untagged yeast extract containing serial dilutions of purified Myc9-tagged Cdc13 protein ranging from 0.5 to 10 femtomoles were run on a gel along with whole cell extracts from the triply tagged strain (Figure 6C). Comparison of the signals of Myc9-tagged Est proteins to the known standards allowed us to determine that there are 1.18±0.32×10^−22^, 0.62±0.11×10^−22^, and 1.40±0.22×10^−22^ moles (or 71.1±19.2, 37.2±6.5, and 84.3±13.3 molecules) of Est1, Est2, and Est3 per cell, respectively (Figure 6D). These results were statistically identical to those obtained from singly Myc9-tagged strains (data not shown).

## Discussion

Although Est1 and Est3 were discovered over 15 years ago [7], [8], their exact roles in telomerase-mediated telomere maintenance have been difficult to establish. Evidence from diverse approaches indicates that Est1 has a key role in recruiting telomerase to DNA ends by virtue of its ability to interact with Cdc13 [14], [15], [26], [27]. This step is likely direct as purified Cdc13 and Est1 interact *in vitro*, and this interaction facilitates Est1 association with telomeric DNA (YW and VAZ, in preparation). However, as summarized in the introduction, current data suggest that Est1 also has a telomerase activation function that is poorly understood. Even less is known about the function(s) of Est3 except that it is clearly essential *in vivo*, and its role cannot be bypassed by a variety of fusion proteins (see Introduction).

Using ChIP, we find that while Est3 telomere binding was bi-phasic, occurring in both G1 and late S/G2 phase, late S/G2 binding was 2 to 3 times higher than G1 phase association. Thus, peak Est3 binding correlated with the time in the cell cycle when telomerase is active (Figure 1B). In contrast, Est3 abundance was not cell cycle regulated (Figure 1C, Figure S1C). Even though at least two kinases, Tel1 and Cdk1/Cdc28 are important for telomerase action [39], [40], [42]–[44], [46]–[48], cell cycle regulated telomere binding of Est3 (Figure 1B), as well as Est1 and Est2 [22], is probably not due to their being phosphorylated in a cell cycle dependent manner. By the criterion of phos-tag induced changes in protein mobility, we found no evidence for phosphorylation of Est1 or Est2 (Figure S2), and although Est3 appeared to be phosphorylated, this modification was not cell cycle regulated (Figure 5). In addition, mutation of the single Cdk1/Cdc28 (S56A) or the single Tel1 (S96A) consensus site in Est3 did not affect telomere length or senescence (CTT and VAZ, data not shown). Therefore, we found no evidence that phosphorylation of Est1, Est2, or Est3 is important for their telomere functions.

We also examined Est3 telomere binding in the absence of other telomerase components. Est3 telomere binding was at background levels in both *tlc1*Δ and *est2*Δ cells (Figure 1B) and very low in *est1*Δ cells (Figure 2B), even though none of these mutations affected Est3 abundance (Figure 1D, Figure 2C). Neither Est1 nor Est2 is telomere associated in *tlc1*Δ or *est2*Δ cells [24]. Therefore, the lack of Est3 telomere binding in these backgrounds could be due to the absence of either Est1 or Est2. However, in *est1*Δ cells, Est2 telomere binding in G1 phase is at wild type levels and reduced but still high (40–50% of wild type binding) in late S/G2 phase [24]. The simplest explanation for these data is that an Est1-Est3 interaction is critical for Est3 telomere binding, especially in late S/G2 phase, since Est3 telomere binding was very low in the absence of Est1, even in situations where there are substantial levels of telomere associated Est2. This interpretation is particularly appealing given our demonstration that purified Est1 and Est3 interact *in vitro* (Figure 3B). Together, these results provide one of the most important mechanistic implications of our data because they suggest that the activation function of Est1 is due to its recruitment of Est3 to telomeres, as proposed previously [30].

Indeed structural considerations have led to the proposal that Est3 is a homologue of the mammalian TPP1 protein [32], [33]. Human TPP1 cooperates with POT1, the human G-strand telomere binding protein, to increase telomerase processivity [35], [36] It has been proposed that the direct interaction between Est3/TPP1 and the G-strand binding protein was lost in *S. cerevisiae*, requiring a new link, Est1, to allow Cdc13-Est3 cooperation [49]. Our data support this hypothesis. Recent *in vitro* data using *S. castellii* Est3 are also consistent with Est3 having a positive effect on telomerase processivity [31]. However, a role for Est3 in promoting processivity is not sufficient to explain all of the Est3 data as Est3 activity seems to be required for even a minimal level of telomere repeat addition *in vivo*. In the case of the mammalian system, in the absence of TPP1, POT1 alone inhibits telomerase activity *in vitro* [35], [50]. Thus, like TPP1, Est3 may function in facilitating telomerase to overcome the inhibition by Cdc13 [51], explaining the complete lack of telomerase activity in the *est3*Δ cell.

Although our data argue that Est1 is the main factor recruiting Est3 to telomeres, our results also suggest that there is a secondary pathway for Est3 recruitment, which is likely Est2-mediated. A minor role for Est2 in Est3 recruitment can explain the low levels of Est3 at telomeres in G1 arrested cells (Figure 1, Figure 2), when Est2 binding is high but Est1 telomere binding is not detected [22]. It can also explain why Est3 was found at low but detectable levels at telomeres in both G1 and late S/G2 phase *est1*Δ cells (Figure 2B). In support of this interpretation, while the Est3 binding at the 60 min time point at both telomeres was reduced over 80% in *est1*Δ cells, telomere binding was reduced only 30% at both telomeres in G1 arrested cells (0 time point) in this background (Figure 2B). Genetic evidence provides strong support for interaction between the TEN domain of Est2 and Est3, although a direct interaction has not yet been established [52]. Our data can also help resolve a discrepancy where one group finds that Est3 association with the holoenzyme is Est1 dependent [25] and one finds that it is Est2, not Est1, dependent [9], [31]. Our data argue that Est1 has a key role and Est2 a more minor role in recruiting Est3 to telomeres (Figure 1, Figure 2). Unlike our telomere binding studies which used synchronous cultures, these other studies were done with asynchronous cells. However, the Est1/Est3 interaction that brings Est3 to telomeres in late S/G2 phase occurred in a relatively narrow window of the cell cycle (Figure 2B), and it is easy to imagine that this dependence could be missed in asynchronous cells. It is possible that the study that found that Est1 was not needed for Est3 to co-immunoprecipitate with TLC1 RNA had a larger fraction of G1 phase cells than in the other study, and therefore, the Est3-TLC1 interaction they detect is Est2, not Est1 mediated.

Another key mechanistic finding from our study is that Est3 acts downstream of both Est1 and Est2. This interpretation is based on the finding that the temporal and quantitative patterns of both Est1 (Figure 4B) and Est2 (Figure 4E) telomere association were not altered in *est3*Δ cells. These findings indicate that Est3′s essential role in telomere maintenance is not to support telomerase binding to Cdc13 coated telomeric DNA, although it might be needed for correct positioning or engagement of the holoenzyme at the very end of the G-tail. Thus, the presence of normal levels of telomere bound Est2/TLC1 RNA, the catalytic core of telomerase, at the appropriate time in the cell cycle, is not sufficient *in vivo* for telomere maintenance even though it supports telomerase action *in vitro*.

Finally our analysis of the abundance of the three Est proteins puts limits on models for how Est3 regulates telomerase. Previous studies that monitored the levels of all yeast proteins as fusions to a GFP or TAP tag [53] detected no signal for any of the Est proteins. Indeed the only core subunit of yeast telomerase whose abundance is known is TLC1, the telomerase RNA, which is estimated to be present in 29.9±3.6 molecules per haploid cell [54]. By using a strain expressing Myc9 tagged versions of Est1, Est2, and Est3, we confirmed that all three Est proteins were present in low amounts, with Est2 being the least (∼37) and Est3 the most (∼84 molecules per cell) abundant (Figure 6D). Telomeres in the triply tagged strain were short but stable (Figure 6A), and the protein levels obtained from this strain were indistinguishable from those obtained in three strains where only one of the three Est proteins was Myc-tagged, and telomere length was less affected (YW and VAZ, data not shown). Therefore, these low abundances are probably not an artifact of partially active subunits, although we cannot rule out this possibility. So far Est2 is the only subunit whose abundance is known to be lower in the absence of another subunit (TLC1) [22] while Est1 is the only subunit whose abundance is cell cycle regulated [22], [25]. Even if Est3 acts as a dimer, as suggested by an earlier study [55], Est3 is probably not the limiting protein subunit. This interpretation is supported by the effects of subunit over-expression on telomere length. While Est3 over-expression does not cause telomere lengthening [52], [56], Est1 over-expression does [56], [57]. These effects can be explained by Est1 dependent, cell cycle and concentration limited recruitment of Est3 to telomeres with a resulting increase in telomerase processivity. Thus, the protein abundance data presented here make it clear that Est3′s unique and essential role in telomerase mediated telomere lengthening is unlikely due to its being the limiting telomerase component.

## Materials and Methods

### Yeast strains

All experiments, unless noted otherwise, were conducted in the YPH background [58] (see Table S1 for strain list). For cell cycle synchrony and chromatin immuno-precipitation (ChIP) experiments, the *BAR1* gene was deleted and replaced with *KanMX6*. All epitope tagged genes were expressed from their own promoters at their endogenous loci. Epitope tagged *EST1* and *EST2* were previously described [22], [23], [37]. Est3 was similarly tagged at its carboxyl terminus with a flexible linker and 9 or 18 Myc epitopes. Telomere lengths were stable and cells did not senesce in either *EST3-G8-MYC9* or *Est3-G8-MYC18* cells but telomeres were shorter than WT in both strains (Est3-G8-Myc9, 50-75 bp shorter and 75 to 125 bps shorter in Est3-G8-Myc18; Figure S1A). Although both Est3- G8-Myc tagged proteins were detectable in whole cell westerns (Figure S1B), only Est3-G8-Myc18 telomere binding was reliably detected by ChIP. Therefore, the *EST3-G8-MYC18* allele was used for ChIP (Figure 1B, Figure 2B) and the *EST3-G8-MYC9* allele was for westerns (Figure 1C, Figure 5, Figure 6). Both chromosomal constructs were verified by sequencing, and thus, the larger apparent molecular weight of the fusion proteins in SDS-PAGE was not due to incorrectly fused proteins. In addition, the tagged loci segregated 2 2 with the *TRP1* marker used to select its integration into the genome, indicating that an unknown protein was not accidently tagged. The *est1*Δ, *est2*Δ and *tlc1*Δ mutations were complete gene deletions and were generated as heterozygous diploids expressing *EST3-G8-MYC18* tagged protein. Likewise, the *est3*Δ mutation was generated in a heterozygous diploid expressing *EST1-MYC9* or *EST2-G8-MYC18* tagged proteins. For all experiments in telomerase deficient strains, newly dissected *est*Δ or *tlc1Δ* spores were replica plated to verify the genotype and then cultured for immediate use so that analysis could be done before cells began to senesce.

### Cell cycle synchrony and ChIP

Cell cycle synchrony experiments were carried out as previously described [22], [23], [39]. Briefly, cells were cultured in rich media to an OD_660_ ∼0.15 and arrested with 0.01 µg/ml alpha factor for 3.5 hr at 24°C with shaking. Aliquots of cells were removed from the alpha factor arrested culture (0 min time point) and at 15 min intervals after release from G1 arrest and processed for fluorescence-activated cell sorting (FACS) analysis and ChIP at each time point. ChIP was performed as described [22], [23], [39] and quantitated on an iCycler iQ Real-Time PCR detection system (Bio-Rad Laboratories). The relative fold enrichment of a protein with telomeres was determined by (TEL_IP_/TEL_IN_)/(ARO1_IP_/ARO_IN_), where IP is the amount of DNA sequence that was amplified from the anti-Myc immuno-precipitate and IN is the amount of DNA sequence that was amplified in the input DNA prior to immuno-precipitation. Each synchrony was repeated at least three times; error bars represent one standard deviation. Where applicable, a two-tailed Student's t test was used to determine statistical significance (P values ≤0.05 were considered significant).

### Western blots

Whole cell extracts from epitope tagged strains were probed with an anti-Myc monoclonal antibody (9E10, Clontech) as previously described [22], [23], [39]. Briefly, cells were grown in rich medium to mid-log phase for asynchronous cell growth or to early log phase as described for cell cycle synchrony. Cells were pelleted, resuspended in CE lysis buffer (50 mM HEPES pH 7.5, 140 mM NaCl, 1 mM EDTA pH 8.0, 10% glycerol, 0.1% IGEPAL CA-630, 1 mM DTT, 1 mM PMSF and 1 tablet protease inhibitor EDTA-free/10mL) and frozen in liquid nitrogen. Cells were thawed quickly then lysed for 1 min by mechanical disruption with the addition of 425–600 µm glass beads using a beat beater at 4°C. Total cell extracts were pre-cleared by centrifugation at 10,000 g for 30 min at 4°C. Protein concentration was determined by Coomassie Plus protein reagent (Pierce) and equivalent protein levels were separated in an 8% SDS-PAGE gel. Proteins were transferred to Immobilon PVDF (0.45 µM) membranes (Millipore), probed with an α-Myc primary antibody and goat-anti-mouse-HRP secondary antibody, and exposed to film.

### Protein purification

Myc9-tagged Cdc13 and Est1 were purified from yeast BCY123 carrying an *arc1-K86R* mutation. Cdc13-Myc9 and Est1 were cloned into a pYES2 vector (Invitrogen) with a carboxyl terminal tag consisting of a G8 linker, 5x Strep-Tag II, and a HAT tag (Clontech). Protein over-expression was induced with 2% galactose at 30°C for 12 hr.

Cdc13-Myc9 was purified by 0.1% polyethyleneimine precipitation, streptactin agarose (Novagen), and Talon Metal Affinity resin (Clontech) and was concentrated and buffer exchanged to TDEG/100 buffer (25 mM Tris-Cl, pH 7.5, 0.1 mM DTT, 0.1 mM EDTA, 10% glycerol, 100 mM NaCl) on an Amicon Ultra-4 (MWCO 50 kDa) concentrator. Concentration was determined using an extinction coefficient of 87,050 M^−1^cm^−1^ at 280 nm.

Est1 was purified by 0.1% polyethyleneimine precipitation, 45% ammonium sulfate precipitation, and a streptactin column. Fractions from the streptactin column were pooled and buffer exchanged to Est1 storage buffer (25 mM Na-HEPES, pH 7.0, 200 mM NaCl, 0.1 mM EDTA, 0.1 mM DTT, 0.05% Triton X-100, 20% glycerol) using a PD-10 column (GE Healthcare). Protein concentration was determined by comparison to known quantities of tagged Cdc13 in western blot analysis against anti-streptag II antibody (Novagen).

Est3 was corrected for its natural +1 frameshifting [59] and cloned into a pET21d vector (Novagen) fused to an amino terminal tag consisting of a HAT tag, 4x streptag II, a G8 linker, and a HRV 3C site. Fresh *E. coli* Rosetta2(DE3) (Novagen) transformants were grown at 18°C, and protein over-expression was induced with 0.1 mM isopropyl β-D-1-thiogalactopyranoside for 24 hr. Cells were lysed by sonication and Est3 was purified by streptactin and Talon columns. The N-terminal Strep-tag was cleaved off by HRV 3C protease and the tag-removed Est3 was concentrated-buffer exchanged to TDEG/100 buffer using an Amicon Ultra-4 (MWCO 10 kDa) concentrator. Protein concentration was determined using an extinction coefficient of 37,410 M^−1^ cm^−1^ at 280 nm.

### Magnetic beads pull-down

A complete reaction contained 1 µM each of Est1 and Est3 in 20 mM Tris-HCl, pH 7.5, 100 mM NaCl, 0.2% Triton X-100, 20 µg/mL BSA, and protease inhibitors (Roche). Reactions were pre-incubated for 15 min on ice before addition of Dynabeads streptavidin M280 (Invitrogen). Reactions were incubated with the beads on a rotary shaker at 4°C for 30 min before separating the unbound from bound proteins on a magnet. The beads were washed 3 times with 200 µl of washing buffer (20 mM Tris-HCl, pH 7.5, 50 mM NaCl, 0.2% Triton X-100, 5% glycerol) before being resuspended in 20 µl loading buffer (36 mM Tris HCl, pH 6.9, 1% SDS, 1% β-mercaptoethanol, 6% glycerol, 0.05% bromophenol blue), boiled, and loaded onto a 10% SDS-PAGE gels along with 15% of input materials. The gel was visualized by Coomassie brilliant blue staining.

### Phos-tag westerns

Extracts were prepared from cells expressing either EST1-Myc9, Myc9-EST2, or EST3-G8-Myc9 that were grown asynchronously at 30°C until an OD_660_ of 0.5 or synchronized as described above. Extracts were prepared by TCA precipitation as described [60]. Briefly, ∼10 mL of culture was centrifuged and washed once with 20% TCA. Cells were resuspended in 20% TCA, glass beads (400-600 µm) were added and cells were lysed in a bead beater for 2 minutes at 4°C (FastPrep 96; MPBiomedical). Extracts were pre-cleared by centrifugation at 3,000 rpm for 10 minutes at 4°C. The pellets were resuspended in buffer and run on western gels. Extracts from cells expressing either Est1 or Est2 were separated on 7.5% SDS-PAGE with or without acrylamide-pendant Phos-tag (Phos-tag AAL-107, Wako) [45]. Extracts from cells expressing Est3 were separated on 10% SDS-PAGE with or without Phos-tag. Two different positive controls were used: *S. pombe* cells expressing Chk1-3HA treated (or not) with 40 µM Camptothecin to induce Chk1 phosphorylation or *S. cerevisiae* cells expressing Rfa1-Myc13 UV irradiated (or not) using a Strategene Stratalinker 1800 at 60 J/m^2^. HA monoclonal antibody (Santa Cruz) was used to detect Chk1. The α-Tubulin loading control was detected with monoclonal antibody (Abcam).

### Quantitative Western blots

Cells were grown in rich medium to early-log phase, briefly sonicated, and the cell density determined on a Beckman Coutler Z2 cell counter. To achieve complete protein extraction, whole cell extracts were prepared as described [61]. Indicated amount of purified standard protein (10, 6, 4, 2, 1, and 0.5 femtomoles) was mixed with extracts from 2×10^7^ cells of untagged strain and heated at 95°C for 3 min before loaded alongside with extracts from 4, 2, 1×10^7^ cells of the triply tagged strain on a 9% SDS-PAGE for anti-Myc Western blot analysis. The tagged proteins were detected using chemiluminescence by a FluroChem CCD camera and quantified by AlphaView software (Alpha Innotech).

## Supporting Information

Figure S1 (A) Telomere length analysis of strains expressing Myc-tagged Est3. Methods are the same as in Figure 6A except samples were resolved in a 1.2% TBE agarose gel. Genomic DNA was prepared from two biological replicates of the indicated strains. To determine if the epitope tagged proteins could support telomere length, freshly dissected spores from hetrozygous diploids (untagged/tagged) were streaked onto solid media every 48 hours for ∼300 generations (12 re-streaks) without evidence for senescence of cells expressing either Myc-tagged Est3 (data not shown). (B) Anti-Myc western blots of cells expressing Est3-G8-Myc9 and Est3-G8-Myc18 (upper panel). An otherwise isogenic untagged strain was loaded alongside as a control. Loading control (LC) of anti-α-tubulin western blots is shown in the bottom. (C) Anti-Myc western blot of chromatin immunoprecipitated Est3-G8-Myc18 from a representative synchrony.
(TIF)

Figure S2 No evidence for phosphorylated Est1 or Est2 is detected throughout a synchronous cell cycle. Cells expressing Myc-tagged Est1(left) or Est2 (right) were synchronized by arrest in alpha factor (0 min time point), extracts prepared at the indicated minutes after release from alpha factor arrest, and separated on 7.5% polyacrylimide gels containing 25 µM phos-tag. Extracts were also prepared from asynchronous cells expressing Myc13-Rfa1 [64] that had been treated with (+) or without (−) 60 J/m^2^ UV. The membranes were reprobed with α-tubulin antibody as a loading control.
(TIF)

Table S1 Yeast strains used in this study.
(DOC)
