# Supplementary figures and images for: The Saccharomyces cerevisiae Telomerase Subunit Est3 Binds Telomeres in a Cell Cycle– and Est1–Dependent Manner and Interacts Directly with Est1 In Vitro
Source: PLoS Genet. 2011 May 5;7(5):e1002060. doi: 10.1371/journal.pgen.1002060 (PMC3088721; doi:10.1371/journal.pgen.1002060)

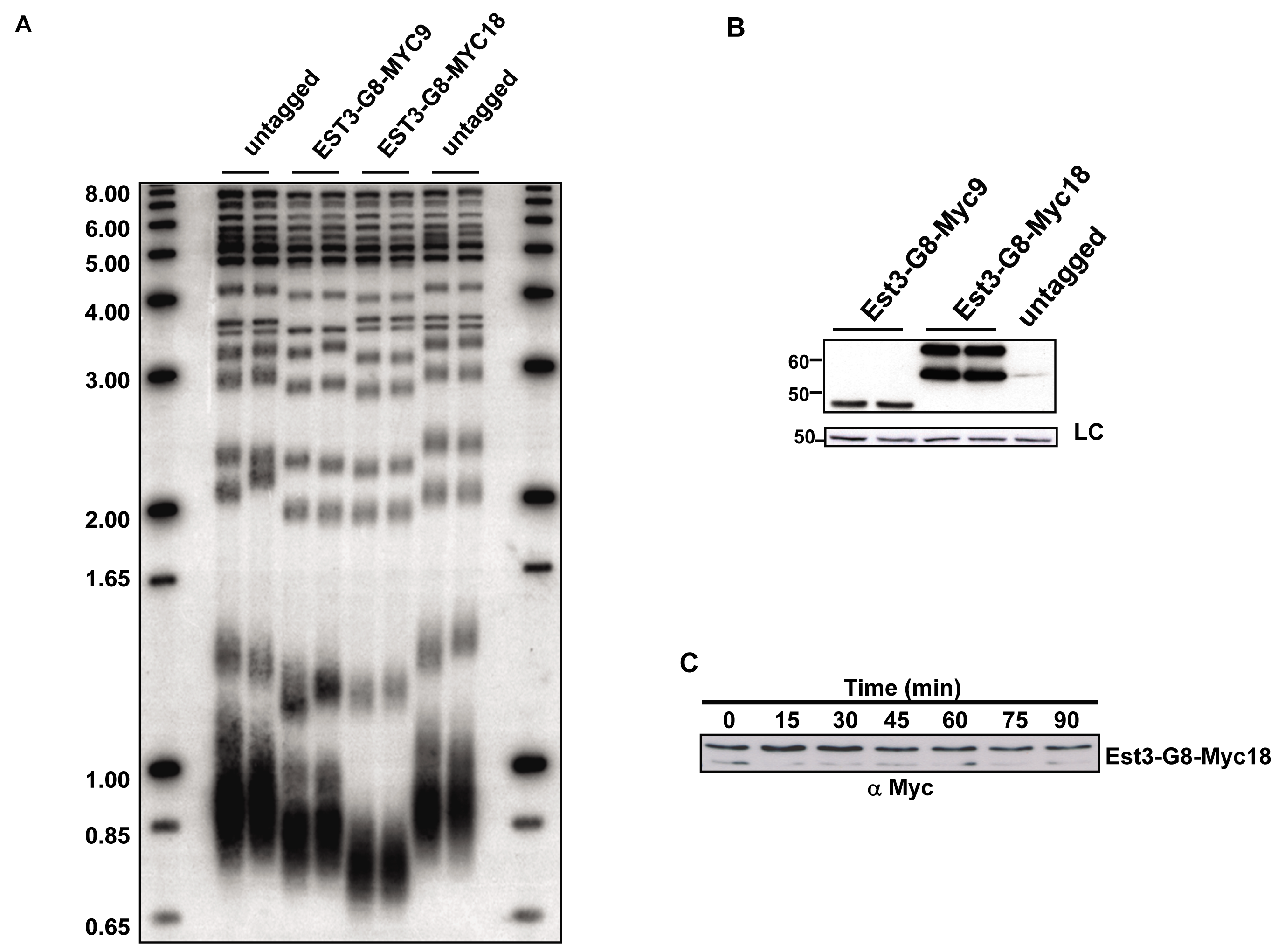

Supplement: Figure S1 — (A) Telomere length analysis of strains expressing Myc-tagged Est3. Methods are the same as in Figure 6A except samples were resolved in a 1.2% TBE agarose gel. Genomic DNA was prepared from two biological replicates of the indicated strains. To determine if the epitope tagged proteins could support telomere length, freshly dissected spores from hetrozygous diploids (untagged/tagged) were streaked onto solid media every 48 hours for ∼300 generations (12 re-streaks) without evidence for senescence of cells expressing either Myc-tagged Est3 (data not shown). (B) Anti-Myc western blots of cells expressing Est3-G8-Myc9 and Est3-G8-Myc18 (upper panel). An otherwise isogenic untagged strain was loaded alongside as a control. Loading control (LC) of anti-α-tubulin western blots is shown in the bottom. (C) Anti-Myc western blot of chromatin immunoprecipitated Est3-G8-Myc18 from a representative synchrony. (TIF) [file pgen.1002060.s001.tif]

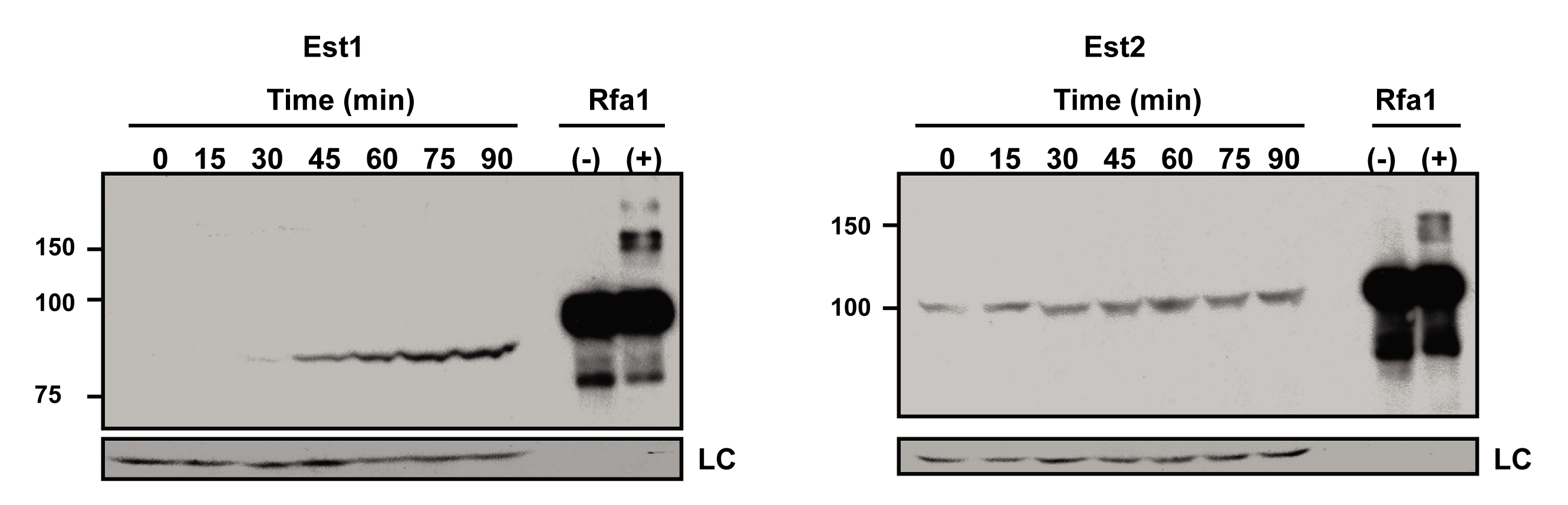

Supplement: Figure S2 — No evidence for phosphorylated Est1 or Est2 is detected throughout a synchronous cell cycle. Cells expressing Myc-tagged Est1(left) or Est2 (right) were synchronized by arrest in alpha factor (0 min time point), extracts prepared at the indicated minutes after release from alpha factor arrest, and separated on 7.5% polyacrylimide gels containing 25 µM phos-tag. Extracts were also prepared from asynchronous cells expressing Myc13-Rfa1 [64] that had been treated with (+) or without (−) 60 J/m2 UV. The membranes were reprobed with α-tubulin antibody as a loading control. (TIF) [file pgen.1002060.s002.tif]
