# Supplementary material for: The Saccharomyces cerevisiae Telomerase Subunit Est3 Binds Telomeres in a Cell Cycle– and Est1–Dependent Manner and Interacts Directly with Est1 In Vitro
Source: PLoS Genet. 2011 May 5;7(5):e1002060. doi: 10.1371/journal.pgen.1002060 (PMC3088721; doi:10.1371/journal.pgen.1002060)
Supplement: Table S1 — Yeast strains used in this study. (DOC) [file pgen.1002060.s003.doc]

| **Strain** | **Genotype** | **Figure** |
| --- | --- | --- |
| yCTT3 | *MATa/α ura3052/ura3052 lys2-801/lys2-801 ade2-101/ade2-101 trp1-Δ63/trp1-Δ63 his3-Δ200/his3-Δ200 leu2-Δ1/leu2-Δ1* |  |
| KMD-J7 | yCTT3 *EST3-G8-MYC18-TRP1/+ est2::HIS3/+ bar1::kanR/+* | 1A-C |
| KMD-B8 | yCTT3 *EST3-G8-MYC18-TRP1/+ tlc1::HIS3/+ bar1::kanR/+* | 1A-C |
| KMD-G7 | yCTT3 *EST3-G8-MYC18-TRP1/+ est1::HIS3/+ bar1::kanR/+* | 2A-C |
| KMD-C4 | yCTT3 *EST1-MYC9-TRP1/+ est3::HIS3/+ bar1::kanR/+* | 3A-C |
| KMD-F5 | yCTT3 *EST2-G8-MYC18-TRP1/+ est3::HIS3/+ bar1::kanR/+* | 3D-F |
| yCTT370 | yCTT3 *EST1-MYC9/+ MYC9EST2/+ EST3-G8-MYC9-TRP1/+ bar1::kanR* | 4 |
| yCTT382 | YPH499 *EST3-G8-MYC9-TRP1 TEL-VII::URA3 bar1::kanR* | 4,5 |
| 1-3D | yCTT382 Est1-MYC9 MYC9-EST2 | 6 |
| yCTT373 | YPH499 *EST1- MYC9 bar1::kanR TEL-VII::URA3* | Sup.3 |
| yCTT381 | YPH499 *Myc9-EST2 bar1::kanR TEL-VII::URA3* | Sup.3 |
| BCY123 *arc1-K86R* | *MATa CAN1 ade2 trp1 Ura3-52 his3 leu2-3, 112 pep4::HIS3, prb1::LEU2 bar1::HisG lys2::pGAL1/10-GAL4 arc1-K86R* | Protein purification |
